# Ectopic expression of the cation-chloride cotransporter KCC2 in blood exosomes as a biomarker for functional rehabilitation

**DOI:** 10.3389/fnmol.2025.1522571

**Published:** 2025-02-05

**Authors:** L. Caccialupi Da Prato, A. Rezzag Lebza, A. Consumi, M. Tessier, A. Srinivasan, C. Rivera, J. Laurin, C. Pellegrino

**Affiliations:** ^1^Inmed, INSERM, Aix-Marseille University, Marseille, France; ^2^Division of Nanoscience and Technology, School of Life Sciences, Center of Excellence in Molecular Biology and Regenerative Medicine, JSS Academy of Higher Education and Research, Mysore, India; ^3^Neuroscience Center, University of Helsinki, Helsinki, Finland

**Keywords:** biomarker, traumatic brain injury, chloride homeostasis, potassium chloride cotransporter 2 (KCC2), exosome

## Abstract

**Background:**

Traumatic brain injury (TBI) is a major cause of disabilities in industrialized countries. Cognitive decline typically occurs in the chronic phase of the condition, following cellular and molecular processes. In this study, we described the use of KCC2, a neuronal-specific potassium–chloride cotransporter, as a potent biomarker to predict cognitive dysfunction after TBI.

**Methods:**

Using neuronal and total exosome collections from the blood serum of the controls and patients with TBI, we were able to anticipate the decline in cognitive performance.

**Results:**

After TBI, we observed a significant and persistent loss of KCC2 expression in the blood exosomes, which was correlated with the changes in the network activity and cellular processes such as secondary neurogenesis. Furthermore, we established a correlation between this decrease in KCC2 expression and the long-term consequences of brain trauma and identified a link between the loss of KCC2 expression and the emergence of depressive-like behavior observed in the mice.

**Conclusion:**

We successfully validated our previous findings, supporting the potential therapeutic benefits of bumetanide in mitigating post-traumatic depression (PTD) following TBI. This effect was correlated with the recovery of KCC2 expression in the blood exosomes, the prevention of extensive neuronal loss among the interneurons, and changes in secondary neurogenesis.

## Introduction

According to the World Health Organization (WHO), traumatic brain injury (TBI) is the leading cause of disability worldwide, with a high incidence in developed countries (Meyer et al., 2008; Bondi et al., 2015). TBI is classified according to multiple factors, such as impaired neurological functions, affected brain areas, and genetic alterations. Long-term consequences of TBI include post-traumatic epilepsy (Kelly et al., 2015; Bragin et al., 2016), cognitive dysfunction, and depressive-like behaviors (Peeters et al., 2015; Perry et al., 2016). Mitigating the consequences of TBI is both socially and economically crucial (Kessler et al., 2009) as most individuals struggle to resume a normal life and return to work.

The prevalence of post-traumatic depression (PTD) after TBI presents with a depressed mood, loss of interest or pleasure, sleep or appetite disturbances, and poor concentration (Roddy et al., 2019). PTD is usually associated with reduced cognitive performance and can become chronic, leading to substantial impairments in the ability to perform daily tasks (Louis et al., 2007). Early diagnosis and recovery after TBI are of primary importance. Of the available therapies, very few have demonstrated a meaningful and sustained effect.

When a TBI occurs, brain damage and cell death initially take place, leading to inflammatory/immunologic responses, blood–brain barrier (BBB) breakdown (Agoston and Kamnaksh, 2019; Vigil et al., 2019), and network rearrangements that can lead to epilepsy (Epsztein et al., 2005; Sloviter, 2008; Kourdougli et al., 2017). The chronic phase is characterized by cognitive decline (Santhakumar et al., 2001) and changes in cellular processes such as hippocampal secondary neurogenesis (Ibrahim et al., 2016). These events are observed in both rodents (Goubert et al., 2019) and humans (Sankar and Mazarati, 2010). It has been suggested that changes in GABAergic neurotransmission play a significant role in these events across all brain regions, including the hippocampus and cortical layers, leading to sustained hyperexcitability of neural networks (Avramescu et al., 2009; Hsieh et al., 2016; Chandrasekar et al., 2019). In addition, dysregulation of the GABAergic pathway is associated with impairments in chloride homeostasis in many neurological and psychiatric disorders. Downregulation of the neuronal-specific chloride and potassium cotransporter KCC2 and up-regulation of the chloride importer NKCC1 have been frequently observed (Medina et al., 2014). Changes in the expression of the chloride cotransporters lead to facilitated depolarization, which may affect the generation of physiologically relevant oscillations in brain networks (Rivera et al., 1999; Kahle et al., 2013; Luscher and Fuchs, 2015). Previous results have also shown that chloride homeostasis is involved in cell survival (Pellegrino et al., 2011), the regulation of neurotrophin signaling (Shulga et al., 2008), inflammation (Pin-Barre et al., 2017), and neurogenesis (Tunc-Ozcan et al., 2019; Carli et al., 2020) under both *in vitro* (Shulga et al., 2012) and *in vivo* conditions (Kourdougli et al., 2017). Taken together, these events demonstrate that markers of chloride homeostasis, such as KCC2 and/or NKCC1, could be used as biomarkers of TBI severity.

Biomarkers are defined as biological characteristics related to normal or pathological activity that can be measured in various biological fluids or organs to detect a disease, predict its severity, or evaluate the effectiveness of treatment (Patel et al., 2014). Several studies have demonstrated the role of circulating exosomes as a new source of biomarkers for pathologies such as cancer and metabolic syndrome (Liu and Cao, 2016). Exosomes are nanovesicles, ranging from 30 to 100 nanometers in diameter, secreted by various cells and detected in all biological fluids (Caby et al., 2005). They can transport nucleic acids, proteins, and lipids specific to their cell of origin (Beach et al., 2014). The involvement of exosomes in immunity and intercellular communication suggests great potential for these vesicles as diagnostic and/or prognostic biomarkers in human pathology, especially as an early diagnostic tool for brain injury and its consequences. This is particularly important since cellular and molecular processes often precede the appearance of cognitive decline (Patel et al., 2014; Brites and Fernandes, 2015; Ko et al., 2018; Sharma et al., 2019; Tiwari et al., 2021).

It therefore seems relevant to first demonstrate whether KCC2 is expressed in exosomes and then assess whether its expression changes after TBI. An important issue in the use of biomarkers is the correct identification of their origin. Interestingly, it is possible to distinguish neuronal-specific exosomes from total exosomes based on the proteins that constitute their membrane (Beach et al., 2014; Bahrini et al., 2015; Hashkavayi et al., 2020). Considering the difficulty of inducing brain cells to express different transgenes *in vivo*, we recently developed an innovative approach that can replace viral injection (Scala et al., 2019) and allows us to distinguish between neuronal expression in the central nervous system and peripheral expression. This method allowed us to tag brain proteins, such as KCC2, in the present study. Using a pharmacological treatment known to affect chloride homeostasis could also be relevant for linking TBI and KCC2 contained in exosomes. Bumetanide, a loop diuretic, specifically inhibits NKCC1. By blocking NKCC1, bumetanide reduces intracellular chloride accumulation, potentially restoring the inhibitory function of GABAergic neurotransmission. Studies have suggested that bumetanide may help reduce secondary neuronal damage and excitotoxicity in TBI by modulating chloride homeostasis. In preclinical studies, bumetanide has shown promise in reducing post-traumatic seizures, brain edema, and neuroinflammation.

The purpose of this study was to demonstrate that neuronal KCC2 contained in exosomes can be used as a relevant biomarker after TBI in young adult mice. We demonstrated that KCC2 is expressed in blood serum and highlighted the presence of neuronal KCC2 in circulating exosomes. Furthermore, we showed that after TBI, KCC2 expression decreases in blood exosomes and can be rescued using bumetanide treatment. Finally, these results are consistent with our previous findings regarding the role of bumetanide in preventing depressive-like behavior after TBI. Taken together, our study suggests that KCC2-containing exosomes could act as a potent biomarker for functional post-traumatic rehabilitation.

## Materials and methods

### Stereotaxic procedure

Ten-week-old C57bl6-J mice were housed in an enriched environment at the INMED animal facility, maintained on a 12 h light / 12 h dark cycle with controlled temperature (23 ± 2°C), and food and water were provided ad libitum. A stereotaxic procedure was performed using an aseptic technique. Briefly, 30 min before the surgery, buprenorphine (0.03 mg/kg) was administered intraperitoneally (i.p), after which the mice were anesthetized with 4% isoflurane vaporized in air, along with an additional 0.3% oxygen enrichment. Surgical anesthesia was maintained using 2% isoflurane with 0.3% oxygen, while the mice were positioned in a stereotaxic frame (David Kopf Instruments, Tujunga, CA). The body temperature was maintained at 37 ± 2°C using a heating pad (Harvard Apparatus). The stereotaxic coordinates were selected to allow injection above CA1 (reference to bregma, anteroposterior axis: −1, laterality: −1.2, verticality: −1.5). The skull was drilled, leaving the dura intact. Using a microinjector (micropump 4, WPI), 1.5 microliters were injected at a rate of 100 nL/min with a NanoFil® D needle (WPI). After the injection, the needle was left in place for an additional 15 min to prevent backflow of the liquid. Altogether, the total duration of the surgery did not exceed 40 min. The skin was sutured, and the animals were placed in the post-operative room of the facility. The brains were then either perfused and fixed for immunochemistry or freshly harvested for western blot analysis 1 week after the injection.

### Controlled-cortical impact model (CCI)

A controlled cortical impact (CCI) procedure was performed using an aseptic technique. Buprenorphine (0.03 mg/kg) was injected intraperitoneally (i.p) 30 min before the surgery. The mice were then anesthetized using 4% isoflurane vaporized in air, along with an additional 0.3% oxygen enrichment. During the surgical procedure, anesthesia was maintained with 2 to 2.5% isoflurane in air, along with 0.3% oxygen. The mice were positioned in a stereotaxic frame (David Kopf Instruments). The body temperature was monitored throughout the procedure using a rectal probe and was maintained at 37 ± 2°C with a heating pad (Harvard Apparatus). A unilateral craniotomy was performed over the right parietal cortex, within the boundaries of the bregma and lambda while leaving the dura intact, using a high-speed drill. The CCI procedure was performed using a Leica impactor with the following parameters: tip diameter 3 mm, speed 6 m/s, depth 1.5 mm, and duration 200 msec. The impact perpendicularly compressed the curvature of the sensorimotor cortex. The animals were allowed to recover on the heating pad before their transfer to the post-surgical room. Before the start of the experiments, the animals were randomly assigned to subgroups, namely sham-vehicle +/− BrainFectIN, sham-bumetanide +/− BrainFectIN, CCI-vehicle +/− BrainFectIN, and CCI-bumetanide +/− BrainFectIN. Bumetanide injections were performed i.p twice daily for a one-week period at a 2 mg/kg concentration.

### Blood collection

Each animal was checked daily after the CCI procedure for weight loss and general condition, according to the protocol validated by our local committee (Apafis #2797). For blood collection, we used the mandibular vein to collect blood from the sham animals and animals with TBI, and a heart puncture was performed when a larger volume was needed from the transfected animals. After applying a local sanitizer to the cheek, the mandibular vein was pierced perpendicularly with a lancet. Blood drops were collected in a blood tube, not exceeding the maximum volume. A small pressure was then applied to stop the bleeding. We used an appropriate lancet (blood lancet Nahita FM024/60425012), and the maximum blood volume allowed for collection was adhered to Table 1. For the heart puncture, after deep anesthesia using 4% isoflurane, the animal was placed in the supine position. After applying a local sanitizer to visualize the depression of the xiphoid appendix, we used a 25-gauge needle. The needle was inserted gently into the xiphoid hollow at an angle of 30°–45° toward the top. A larger volume of blood was withdrawn; if the blood did not flow, the needle location was slightly adjusted.

**Table 1:** Maximum blood volume collected according to the puncture sites.

Location of the blood collection	Approximate volume
Mandibular vein	100–200 μL
Cardiac puncture	0.5–1.0 mL

### Total exosome collection

The exosomes were isolated from 100 microliters of the blood serum, after the addition of 5 μL protease and phosphatase inhibitor (Pierce Protease and Phosphatase Inhibitor Mini Tablets, EDTA-FREE, Invitrogen A32961). The total exosomes were first isolated from the blood serum using the “Total Exosome Isolation Reagent (from serum)” (Thermo Fisher Scientific, Invitrogen 4478360) according to the manufacturer’s instruction. After the collection, the exosomes were stored at 2°C to 8°C for up to 1 week or at −20°C for longer-term storage. The neuronal fraction was enriched by immunoprecipitation using the L1CAM antibody (eBio5G3 (5G3), Thermo Fisher Scientific, #14-1719-82) but was probed with CD63 to avoid interfering with the KCC2 staining.

### Immunohistochemistry

The mice were deeply anesthetized with an intraperitoneal injection of ketamine/xylazine and then transcardially perfused with cold phosphate-buffered saline (PBS 0.01 M), followed by a 3% paraformaldehyde solution (AntigenFix, Diapath). The brains were post-fixed overnight in 3% paraformaldehyde at 4°C and then coronally sliced using a Leica VT1200S Vibratome. A total of 60 μm-thick sections were permeabilized and blocked in PBS with 0.3% Triton X-100 and 5% normal goat serum (NGS) for 1 h at room temperature. Then, the sections were stained with primary antibodies diluted in PBS with 5% NGS and 0.1% Triton X-100 at 4°C overnight using anti-Dsred (Takara Bio Clontech, living colors polyclonal antibody, 632496), anti-NeuN (Merck Millipore; MAB377), and anti-Gad67 (Merck Millipore, MAB5406). After washing with PBS, the slices were incubated with the corresponding Alexa Fluor 488 and 555-conjugated secondary antibodies, diluted in PBS (1/500, Thermo Fisher Scientific, Invitrogen A11001), for 2 h at room temperature and finally counterstained for 1 min with Hoechst 33258 (10 μg/mL in PBS, Sigma-Aldrich, 94403). The sections were mounted onto Superfrost Plus glass slides in Fluoromount-G Mounting Medium. For each section, serial images were taken using a fluorescence microscope equipped with an apotome module and 20× or 40× objectives.

### Protein extraction and western blot

The animals were euthanized by decapitation after deep isoflurane anesthesia. The hippocampi were quickly dissected out, flash-frozen in liquid nitrogen, and stored at −80°C. The brain tissues were homogenized in RIPA buffer (50 mM Tris–HCl pH 8, 150 mM NaCl; SDS 0.1%; Deoxycholic Acid 0.5%; 1% Triton X-100) supplemented with a Protease/Phosphatase Inhibitor Tablet (Thermo Fisher Scientific). The proteins were run on a polyacrylamide gel (Bolt 4–12% Bis-Tris plus, Invitrogen by Thermo Fisher Scientific) and transferred to a nitrocellulose membrane (GE Healthcare Life Science). Following the application of a blocking solution containing Tris-buffered saline, 0.1% Tween and 5% bovine serum albumin (BSA), the membranes were incubated overnight at 4°C with primary antibodies diluted in a blocking solution (Tris-buffered saline/ 0.1% tween/ 2.5% BSA): anti-Dsred (Clontech, Living Color Ds-Red Polyclonal Antibody, 632,496), anti-KCC2 [home-made antibody, (Ludwig et al., 2003)], and Tubuline-β3 (Biolegend, 802001). Horseradish peroxidase-conjugated anti-rabbit or anti-mouse IgG (Agilent Dako) was used as secondary antibodies, diluted in 5% bovine albumin at room temperature for 2 h. Bands were then detected using SuperSignal West Pico (Thermo Fisher Scientific, #34080) and analyzed with the G:Box imaging system (Syngene). Quantifications were performed using the Gel Plot Analyzer plugin in ImageJ.

### Drug delivery

A 20 mM stock solution of bumetanide (Sigma-Aldrich, B3023) was prepared by dissolving 36.4 mg of the powder in 1 mL of absolute ethanol. The injected solution was created by mixing 40 μL of the stock solution with 4 mL of 1X PBS. A dose of 26.7 μL per gram of the animal body weight was then injected intraperitoneally (2 mg/kg), twice daily (9 AM and 5 PM). A vehicle solution was prepared using the same procedure but without the bumetanide powder to maintain the volume and diluent.

### Plasmid construct

The KCC2-mCherry construct was created by inserting a ubiquitin promoter in place of the CMV promoter in the pmCherry vector (Clontech) to enhance transgene expression under *in vivo* conditions. The vector and transgene were sequenced before cloning, and the full plasmid was also sequenced.

### Statistical analysis

All mean values were presented with the standard deviation (SD). Normality was tested for each distribution, with a significance level set at 5%. Two-tailed Student’s *t*-test, the Mann–Whitney U test, or one-way ANOVA was used accordingly, using Prism software (GraphPad Software, Inc., La Jolla, CA, United States). Box plots reported the median, interquartile range, and total range of the data, with statistical significance represented as follows: ∗*p* < 0.05, ∗∗*p* < 0.01, and ∗∗∗*p* < 0.001.

## Results

### Is KCC2 detectable in circulating blood?

**Figure 1:**
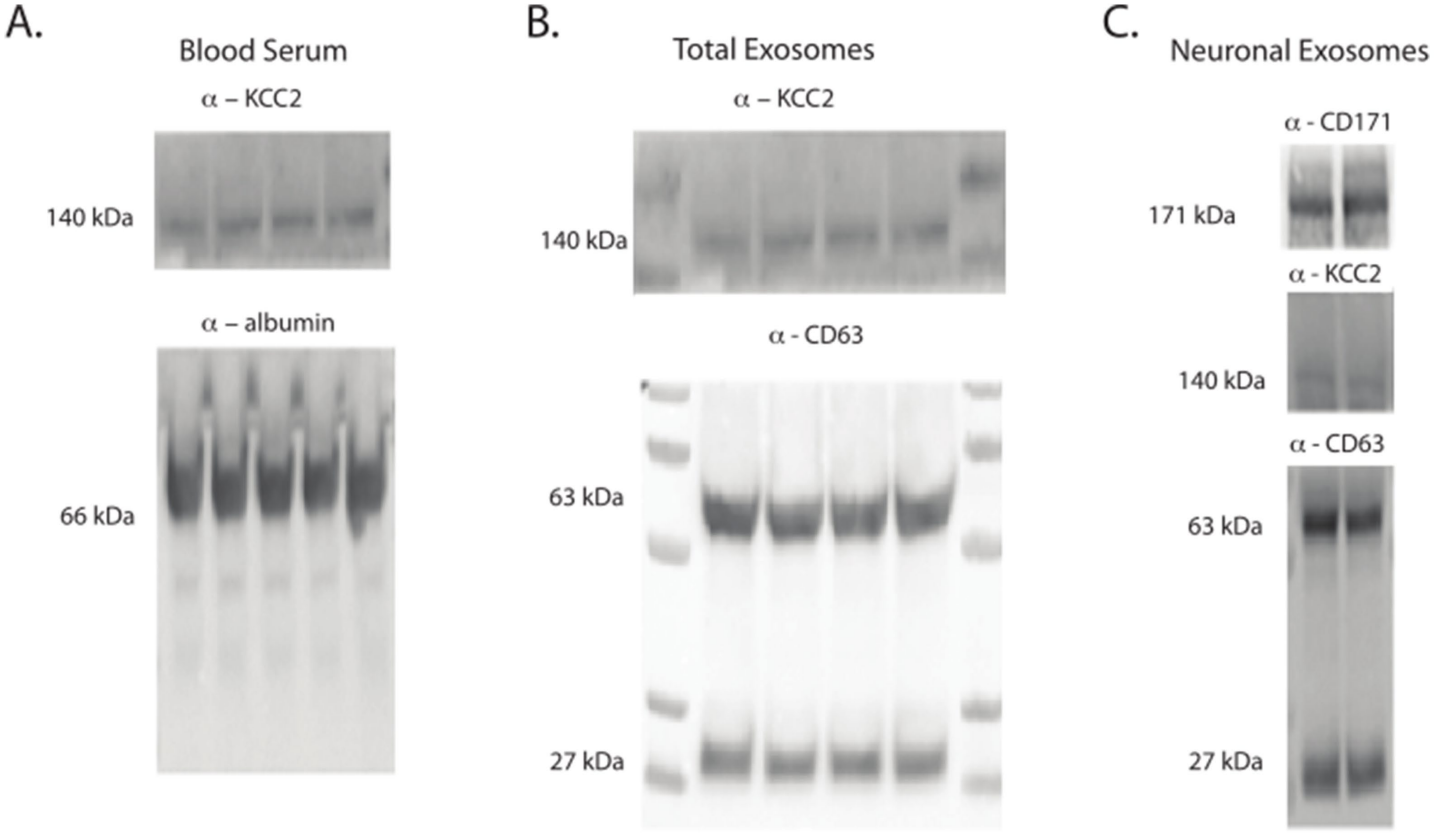
Identification of KCC2. **(A)** KCC2 detected in the Blood serum (140 kDa). **(B)** KCC2 in the total exosomes from the blood shows the same band detected in the serum, confirming that the band corresponds to KCC2. **(C)** KCC2 was identified in both neuronal exosomes and neuronal-derived exosomes purified from the serum, with confirmation of their neuronal origin by detecting the exosomal marker CD171.

Our initial investigation aimed to determine whether KCC2 can be detected in blood serum. A previous study successfully identified it in cerebrospinal fluid (Duarte et al., 2013), making it essential to first ascertain its presence in blood serum. We collected 0.2 mL of blood from the mandibular vein of the mice and isolated the serum by centrifugation at 2,000 *g* for 30 min at 4°C. Subsequently, we conducted a western blot under denaturing conditions using the clarified serum. As shown in Figure 1A, we clearly observed the full-length KCC2 band at 140 kDa in all tested samples (*n* = 30). Utilizing our custom-made antibody specific to the N-terminus of KCC2 (Ludwig et al., 2003), it was challenging to definitively identify degraded forms of KCC2. Nonetheless, after analyzing the gel band profiles with red Ponceau, we found no discernible difference compared to the brain tissue samples (Figure 1B, data not shown). This intriguing result prompted us to investigate whether the secretion of KCC2 into the blood was constitutive or dependent on specific conditions.

### Are KCC2-containing exosomes found in blood serum?

In the subsequent step, we aimed to investigate the presence of KCC2 in the circulating exosomes, originating either from the neuronal cells or from the entire cell population, as these vesicles are known to travel long distances. We utilized a specific extraction kit to purify the total exosomes from the peripheral serum through immunoprecipitation. The isolation of the total exosomes was confirmed by detecting the pan-exosomal marker CD63 (Hashkavayi et al., 2020) in each sample using western blot analysis (Figure 1B). The figure illustrates both the glycosylated form (63 kDa) and non-glycosylated form (27 kDa) of the CD63 protein, with a predominance of the glycosylated form at 63kd.

To confirm the neuronal origin of KCC2, we proceeded with the purification of the neuronal exosomes from the total exosome samples once again, this time using immunoprecipitation. To verify the neuronal origin of KCC2, we conducted western blotting with the neuronal exosome marker L1CAM (CD171) (Pulliam et al., 2019; Figure 1C).

### Do KCC2-containing blood exosomes have a neuronal origin?

**Figure 2:**
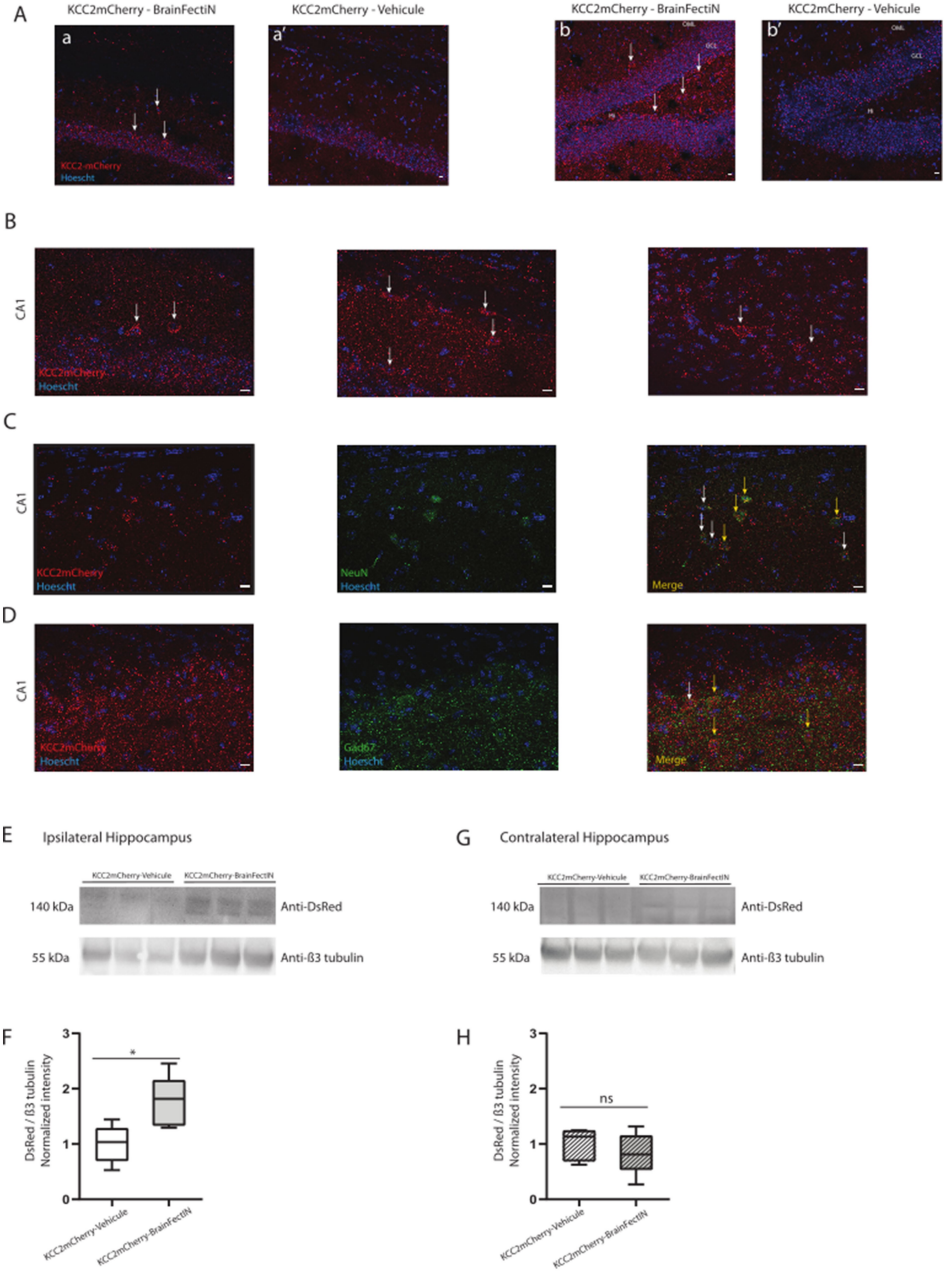
mCherry-tagged KCC2 expression in the hippocampus 1 week after the KCC2-mCherry- BrainFectIN injection. **(A)** Expression of KCC2-mCherry in the ipsilateral CA1 area **(a)** and the ipsilateral dentate gyrus **(b)** (white arrows). The sham mice were injected with the KCC2-mCherry construct and physiologic serum instead of BrainFectIN, showing no positive cells in the CA1 area **(a’)** and the dentate gyrus area **(b’)**. Hi, Hilus; GCL, Granule Cell Layer; OML, Outer Molecular Layer. Scale bar = 10 μM. Magnification 20x. **(B)** Magnification of the injection site in the CA1 area, showing the expression of the mCherry-tagged KCC2. Scale bar = 10 μM. Magnification 40x. **(C,D)** Representation of NeuN **(C)** and Gad67 **(D)** staining of the KCC-mCherry positive cells (yellow arrows). White arrows indicate the lack of co-localization. Scale bar = 10 μM. Magnification 40x. **(E)** Representative western blots of the ipsilateral hippocampi extracted from the KCC2-mCherry-BrainFectIN-injected mice (*n* = 5) and sham mice (*n* = 5). The hippocampi were extracted 1 week after the injection. **(F)** Corresponding western blot quantification from the KCC2-mCherry-BrainFectIN-injected mice and sham mice. The data are presented as median (with interquartile range). The Mann–Whitney U test analysis reported the following: *p < 0.05; n.s., not significant, under Prism analysis. **(G)** Representative western blots of the contralateral hippocampi extracted from the KCC2-mCherry-BrainFectIN-injected mice (*n* = 5) and sham mice (*n* = 5). The hippocampi were extracted 1 week after the injection. **(H)** Corresponding western blot quantification from the KCC2-mCherry-BrainFectIN-injected mice and sham mice. The data are presented as median (with interquartile range). The Mann–Whitney U test analysis reported the following: *p < 0.05; n.s., not significant, under Prism analysis.

KCC2 is primarily expressed in the central nervous system (Blaesse et al., 2009), making the discovery of its full-length form in circulating blood particularly intriguing. To verify the CNS origin of the molecule, we chose to genetically modify the brain neuronal cells through *in vivo* transfection using a BrainFectIN® agent. Using a stereotaxic approach, we injected DNA encoding C-terminal tagged mCherry-KCC2. One week after the injection, we harvested the brains and performed co-immunoprecipitation. Our previous work with BrainFectIN® had already demonstrated potent and sustained modification of CNS cells (Scala et al., 2019). The *post hoc* analysis 1 week after the injection revealed transgene expression through immunohistochemistry, using a specific antibody against the mCherry tag at the injection site, CA1 (Figures 2Aa), and in the dentate gyrus (DG) (Figures 2Ab). High magnification of the CA1 region of the hippocampus (Figure 2B) confirmed the robust expression of the mCherry-tagged KCC2. We further confirmed the neuronal expression of the transgene in the principal neurons using the neuronal marker NeuN (Figure 2C) and in the interneurons using the Gad67 antibody (Figure 2D).

At the protein level, we verified the presence of the mCherry-tagged KCC2 in the ipsilateral hippocampal extract. This region was selected due to its targeting by the injection (1.76 ± 0.20, *n* = 5 vs. 1.0 ± 0.15, *n* = 5, *p* = 0.03, Figures 2E,F). As previously demonstrated by immunohistochemistry, there was no diffusion of the transgene to the contralateral side (0.83 ± 0.17, *n* = 5 vs. 1.00 ± 0.13, *n* = 5, *p* = 0.84, Figures 2G,H).

**Figure 3:**
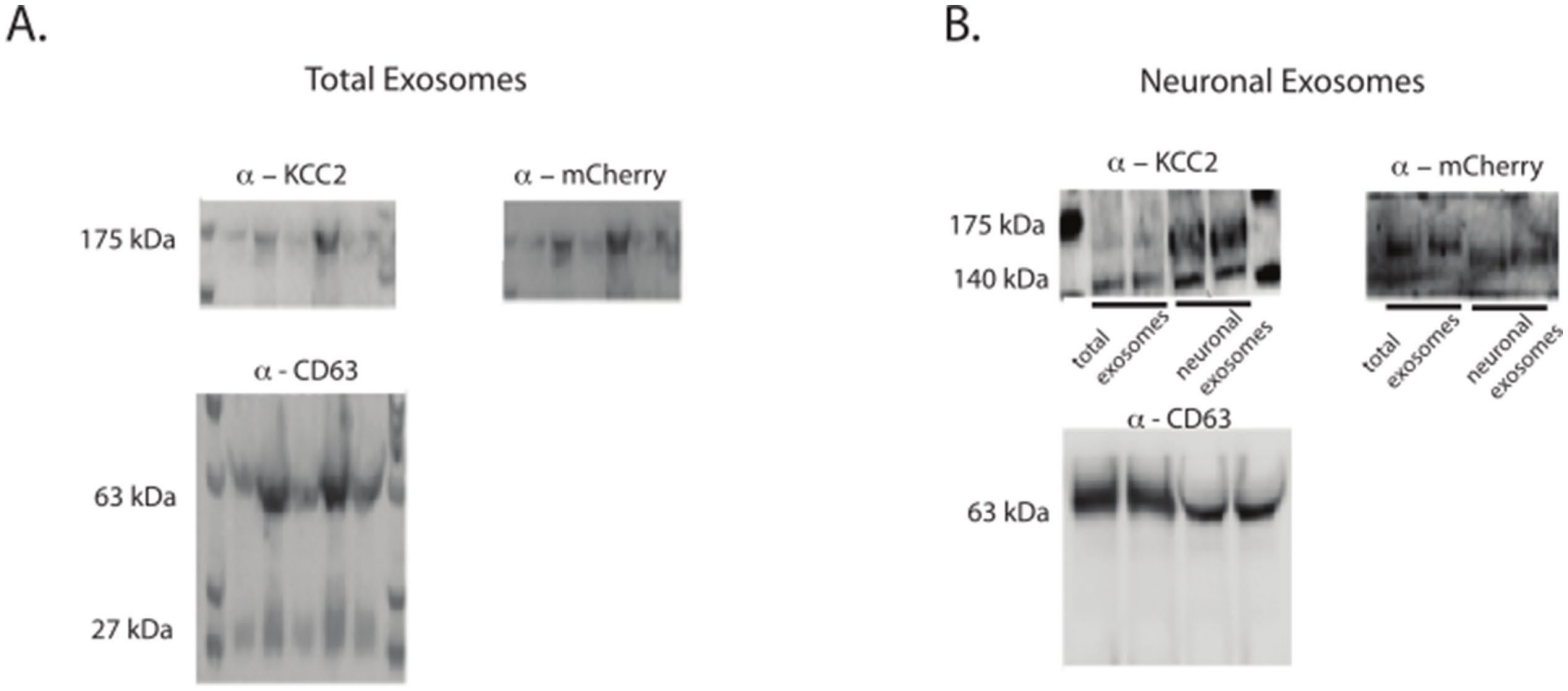
mCherry-tagged KCC2 expression in the exosomes after the BrainFectIN injection. **(A)** Expression of KCC2 and KCC2-mCherry in the total exosomes after the protein extraction. **(B)** Expression of KCC2 and KCC2-mCherry tag in the neuronal exosomes after the protein extraction. Representative western blots of the protein extracted from the KCC2-mCherry-BrainFectIN-injected mice (*n* = 6) and sham mice (*n* = 6). The protein exosomes were extracted 1 week after the injection.

In this context, we were able to identify the 35 kDa-shifted KCC2 band corresponding to the tagged KCC2 in the pan-exosomes CD63-containing exosomes (Figure 3A). The CNS origin was further reinforced and confirmed using L1CAM immunoprecipitation of the neuronal exosomes, confirming the neuronal enrichment of the mCherry-KCC2 (Figure 3B). Furthermore, by combining the KCC2 antibody, which recognizes the N-terminus part of the protein, and the mCherry antibody, which recognizes the mCherry molecule at the C-terminal part of KCC2, we conclusively confirmed that full-length KCC2 was embedded in the exosomes, particularly the neuronal exosomes. The combination of these two antibodies targeted both the N- and C-terminus regions of full-length KCC2, as indicated by the bands at 140 and 175 kDa in Figure 2.

### Does traumatic brain injury (TBI) have an effect on exosome content?

It has been previously proposed and demonstrated that there is a transient loss of KCC2 expression after trauma (Medina et al., 2014). Utilizing the controlled-cortical impact model, we observed a robust decrease in the KCC2 levels in the early days after the trauma, followed by complete recovery after 1 week (Goubert et al., 2019). Considering these findings, we also investigated the impact of TBI on the exosomes, both total and neuronal, in the blood serum using the same paradigm.

**Figure 4:**
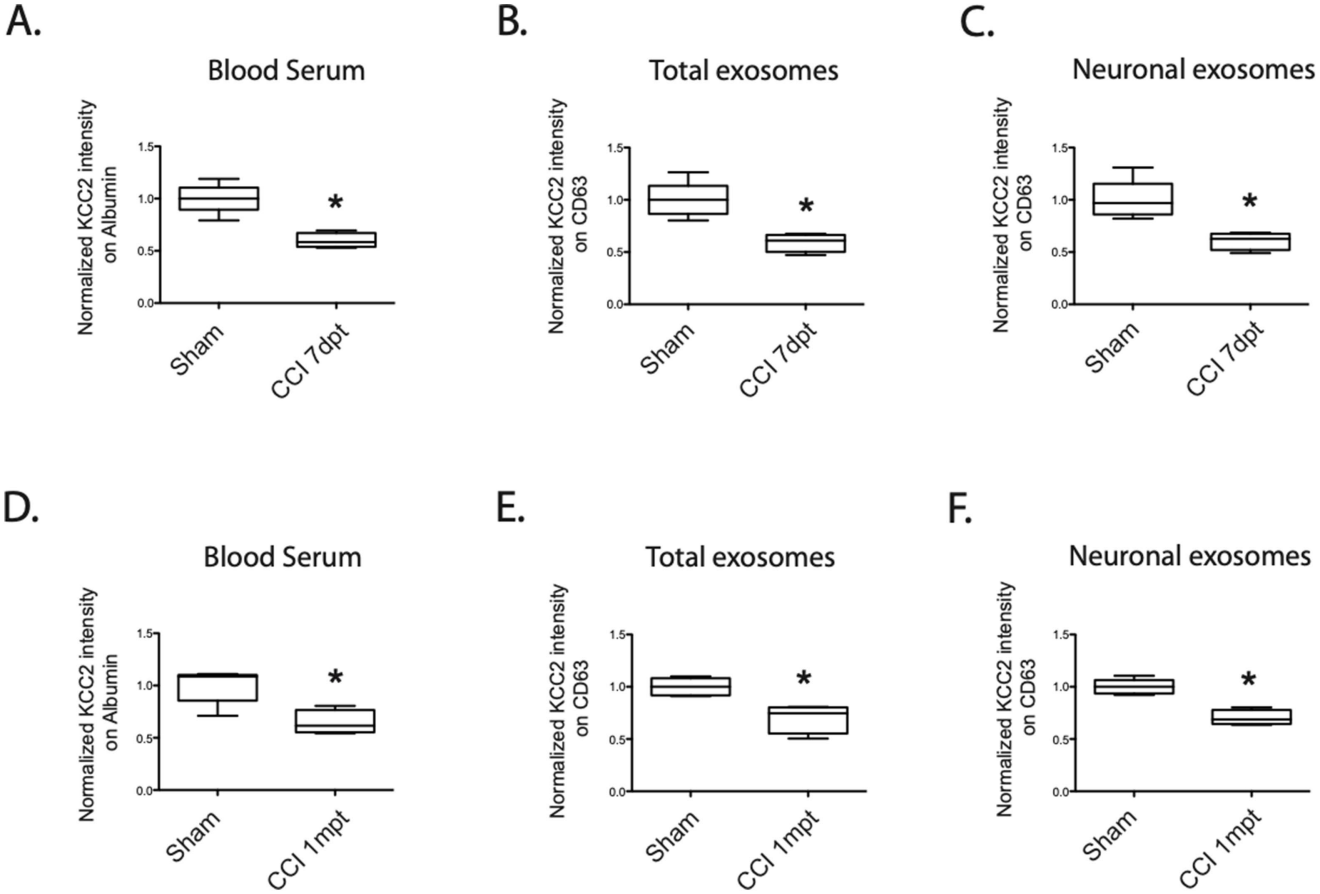
KCC2 expression in the serum, total exosomes, and neuronal exosomes under the CCI condition. **(A)** Expression of KCC2 in the blood serum at 7dpt, sham versus CCI condition, *n* = 4 animals per condition. **(B)** Expression of KCC2 in the total exosomes at 7dpt, sham versus CCI condition, *n* = 4 animals per condition. **(C)** Expression of KCC2 in the neuronal exosomes at 7 dpt, sham versus CCI condition, *n* = 4 animals per condition. **(D)** Expression of KCC2 in the blood serum at 1 mpt, sham versus CCI condition, *n* = 4 animals per condition. **(E)** Expression of KCC2 in the total exosomes at 1mpt, sham versus CCI condition, *n* = 4 animals per condition. **(F)** Expression of KCC2 in the neuronal exosomes at 1mpt, sham versus CCI condition, *n* = 4 animals per condition. The Student’s *t*-test analysis reported the following: *p < 0.05; **p < 0.01; and ***p < 0.001, under Prism analysis.

At 7 days post-trauma (dpt), we clearly observed a significant decrease in the normalized total expression of KCC2 in the blood serum (sham 100% ± 14 vs. CCI 59.8% ± 7, *p = 0.015, *n* = 4, Figure 4A), as well as in the total exosomes (sham 100% ± 16 vs. CCI 59% ± 8, *p = 0.015, *n* = 4, Figure 4B) and the neuronal-enriched fraction of the exosomes (sham 100% ± 18 vs. CCI 60% ± 8, *p = 0.016, *n* = 4, Figure 4C).

Considering the possibility that this loss of expression might persist for a longer duration in the blood compared to the CNS expression, we conducted the same analysis 1 month after the trauma. This timeframe was chosen because we had previously demonstrated that KCC2 expression in the hippocampus recovered to sham levels in the same animal model, while the subjects exhibited cognitive dysfunction, particularly depression-like behavior (Goubert et al., 2019).

Once again, we observed a substantial loss of KCC2 expression in the blood serum (sham 100% ± 8 vs. CCI 64% ± 11, *p = 0.03, *n* = 4, Figure 4D), in the total exosomes (sham 100% ± 8 vs. CCI 70% ± 13, *p = 0.016, *n* = 4, Figure 4E), and in the neuronal exosomes (sham 100% ± 7 vs. CCI 70% ± 7, *p = 0.016, *n* = 4, Figure 4F). These findings support the hypothesis of a sustained and permanent decrease in KCC2 expression in exosomes following TBI.

### Does bumetanide affect exosome content?

Previous research, including studies by Goubert et al. (2019) and Pellegrino et al. (2011), has shown that bumetanide, a sodium–potassium–chloride transporter antagonist, exerts a powerful effect in preventing cell death in interneurons and principal cells. In addition, it has been found to prevent rapid and transient degradation of KCC2 in various trauma models (Hu et al., 2017; Medina et al., 2014) and epilepsy (Kourdougli et al., 2017). Given its ability to efficiently restore KCC2 expression in neuronal cells, we sought to investigate its potential impact on KCC2 modulation in exosome content.

**Figure 5:**
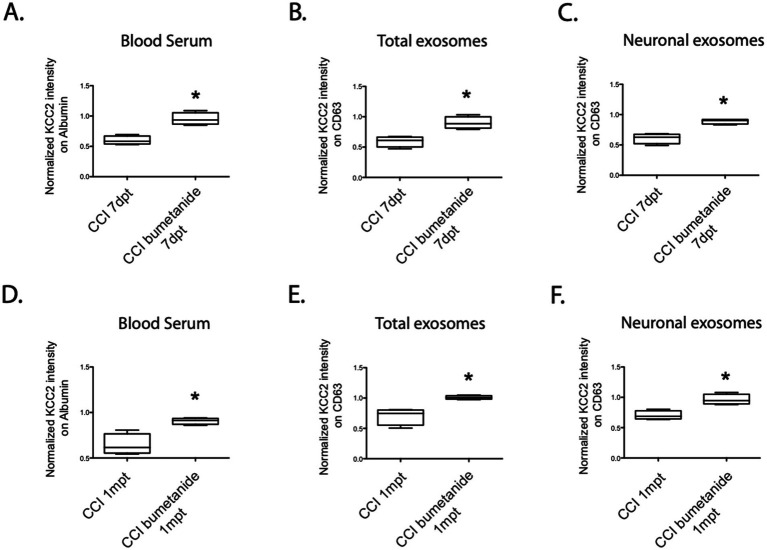
KCC2 expression in the serum, total exosomes, and neuronal exosomes under the CCI condition with the bumetanide treatment. **(A)** Expression of KCC2 in the blood serum at 7dpt, CCI versus bumetanide-treated CCI condition, *n* = 4 animals per condition. **(B)** Expression of KCC2 in the total exosomes at 7dpt, CCI versus bumetanide-treated CCI condition, *n* = 4 animals per condition. **(C)** Expression of KCC2 in the neuronal exosomes at 7dpt, CCI versus bumetanide-treated CCI condition, *n* = 4 animals per condition. **(D)** Expression of KCC2 in the blood serum at 1 mpt, CCI versus bumetanide-treated CCI condition, *n* = 4 animals per condition. **(E)** Expression of KCC2 in the total exosomes at 1 mpt, CCI versus bumetanide-treated CCI condition, *n* = 4 animals per condition. **(F)** Expression of KCC2 in the neuronal exosomes at 1 mpt, CCI versus bumetanide-treated CCI condition, *n* = 4 animals per condition. The Student’s *t*-test analysis reported the following: *p < 0.05; **p < 0.01; and ***p < 0.001, under Prism analysis.

To explore this, we administered intraperitoneal injections of 2 mg/kg bumetanide twice daily for 1 week. Subsequently, we collected blood serum to assess KCC2 expression. One week after the trauma (7dpt), the KCC2 expression levels were significantly improved compared to the CCI condition, resembling the levels seen in the sham group. This improvement was observed both in the blood serum (CCI 59% ± 7 vs. CCIbum 95% ± 10, *p = 0.028, Figure 5A), total exosomes (CCI 59% ± 8 vs. CCIbum 90% ± 9, *p = 0.028, Figure 5B), and neuronal exosomes (CCI 60% ± 8 vs. CCIbum 89% ± 4, *p = 0.028, *n* = 4, Figure 5C).

Later, our focus shifted to the long-term effects of bumetanide on the persistence of KCC2 expression and its correlation with the prevention of depressive-like behavior, which has been previously observed. To investigate whether the treatment could also modify KCC2 expression in the exosomes, we assessed the KCC2 levels in the blood 1 month after the treatment. Notably, we observed differences in KCC2 expression in the blood compared to the CCI condition in the blood serum (CCI 64% ± 11 vs. CCIbum 90% ± 3, *p = 0.028, *n* = 4, Figure 5D), total exosomes (CCI 70% ± 13 vs. CCIbum 1.01% ± 3, *p = 0.028, *n* = 4, Figure 5E), and neuronal exosomes (CCI 70% ± 7 vs. CCIbum 96% ± 8, *p = 0.028, *n* = 4, Figure 5F). These findings suggest that KCC2 expression in exosomes could be a relevant marker for detecting depressive-like behavior, consistent with the functional changes observed in our previous study (Goubert et al., 2019).

## Discussion

Exosomes have been hypothesized to play a pathological role in several neurological disorders, particularly proteinopathies, by spreading pathological molecules to healthy tissue. Alternatively, exosomes have been postulated to play a protective role by transporting pathological molecules out of cells (Quek and Hill, 2017). This is quite an innovative field of research as recent studies have proposed a role for exosomes in inflammation (Brites and Fernandes, 2015) and miRNA regulation. These vesicles may transport molecules over large distances and influence cell function under physiological and pathological conditions (Brites and Fernandes, 2015; Hashkavayi et al., 2020). More recently, exosomes have been hypothesized to play important roles in the nervous system and have been shown to be involved in neurodegenerative disorders (Beach et al., 2014), axonal pathfinding through cell contact (Gong et al., 2016), secondary neurogenesis (Fuller et al., 2020), synaptic pruning (Bahrini et al., 2015), and network assembly (Sharma et al., 2019). However, the potential role of exosomes as routes for transporting brain-specific membrane-embedded proteins in pathological conditions is poorly studied. In this study, we proposed a potential role for exosomes as early biomarkers of brain injury related to depressive-like behavior. This psychiatric disorder has a high incidence after trauma, both in the general population and in military contexts (Lange et al., 2020). Having prognostic tools would be highly valuable for preventing and treating patients at early stages. Furthermore, biomarkers can facilitate the early detection of brain pathologies, improving decision-making regarding therapeutic approaches, which is crucial in treating psychiatric disorders. For instance, the assessment of chloride homeostasis imbalance, specifically KCC2 dysregulation, has already yielded promising results in other pathologies, such as hepatic encephalopathy (HE) (Li et al., 2012). Indeed, HE is defined as a neuropsychiatric disorder resulting from hepatic dysfunction. It has been shown that KCC2 downregulation in the blood serum of cirrhotic patients is not only correlated with HE diagnosis but also that the extent of the NKCC1/KCC2 imbalance may be relevant for assessing the severity of the disease. Hence, investigating the role of KCC2 as a biomarker for brain disorders is crucial.

Many studies have used bumetanide to treat chloride homeostasis imbalance. Although the permeability of the blood–brain barrier can be affected by pathological conditions, it is common for the BBB to exhibit compromised integrity. It is highly likely that such conditions were present during the time window of the bumetanide application in this study (Cohen et al., 2007), facilitating its entry into the brain at pharmacologically relevant concentrations. However, a recent study by Löscher and collaborators described the relevant bumetanide concentration needed to reach the brain (Löscher and Kaila, 2022).

However, although KCC2 is known as a neuron-specific chloride cotransporter, we know now that it is also expressed in structures other than the CNS. Indeed, it has been demonstrated that KCC2 is also expressed in various pancreatic cells. Specifically, KCC2 is found in the glucagon-related alpha cells and, more predominantly, in the beta cells of the pancreatic islet (Kursan et al., 2017). In the pancreas, it has been hypothesized that GABAergic regulation, together with insulin, plays a role in decreasing hyperglycemia (Bansal and Wang, 2008). These findings highlight the necessity of tracking the origins of the protein in circulating blood. To address this issue, we ensured the measurement of neuron-specific KCC2-containing exosomes using the BrainFectIN agent. Interestingly, when assessing the chloride cotransporter expression in the total exosome content, we were not able to detect any difference in the NKCC1 blood-exosome expression between the sham and CCI animals. This confirms that KCC2 is the only effective marker of brain trauma (data not shown). In summary, investigating non-invasive methods, such as the use of biomarkers, as relevant tools for diagnosing various afflictions is essential and could improve the prognosis of several illnesses. Finally, assessing the level of KCC2 in brain-specific exosomes, as described in this study, could be developed into a valuable tool for monitoring the progression of brain trauma associated with different clinical settings.

## Data Availability

The raw data supporting the conclusions of this article will be made available by the authors, without undue reservation.

## References

[ref1] Agoston D. V. Kamnaksh A. (2019). Protein biomarkers of epileptogenicity after traumatic brain injury. Neurobiol. Dis. 123, 59–68. doi: 10.1016/j.nbd.2018.07.017, PMID: 30030023 PMC6800147

[ref2] Avramescu S. Nita D. A. Timofeev I. (2009). Neocortical post-traumatic epileptogenesis is associated with loss of GABAergic neurons. J. Neurotrauma 26, 799–812. doi: 10.1089/neu.2008.0739, PMID: 19422294 PMC2735829

[ref3] Bahrini I. Song J. Diez D. Hanayama R. (2015). Neuronal exosomes facilitate synaptic pruning by up-regulating complement factors in microglia. Sci. Rep. 5:7989. doi: 10.1038/srep07989, PMID: 25612542 PMC4303875

[ref4] Bansal P. Wang Q. (2008). Insulin as a physiological modulator of glucagon secretion. Am. J. Physiol.-Endocrinol. Metab. 295, E751–E761. doi: 10.1152/ajpendo.90295.2008, PMID: 18647881

[ref5] Beach A. Zhang H.-G. Ratajczak M. Z. Kakar S. S. (2014). Exosomes: an overview of biogenesis, composition and role in ovarian cancer. J. Ovarian Res. 7:14. doi: 10.1186/1757-2215-7-14, PMID: 24460816 PMC3932023

[ref6] Blaesse P. Airaksinen M. S. Rivera C. Kaila K. (2009). Cation-chloride cotransporters and neuronal function. Neuron 61, 820–838. doi: 10.1016/j.neuron.2009.03.003 19323993

[ref7] Bondi C. O. Semple B. D. Noble-Haeusslein L. J. Osier N. D. Carlson S. W. Dixon C. E.. (2015). Found in translation: understanding the biology and behavior of experimental traumatic brain injury. Neurosci. Biobehav. Rev. 58, 123–146. doi: 10.1016/j.neubiorev.2014.12.004, PMID: 25496906 PMC4465064

[ref8] Bragin A. Li L. Almajano J. Alvarado-Rojas C. Reid A. Y. Staba R. J.. (2016). Pathologic electrographic changes after experimental traumatic brain injury. Epilepsia 57, 735–745. doi: 10.1111/epi.13359, PMID: 27012461 PMC5081251

[ref9] Brites D. Fernandes A. (2015). Neuroinflammation and depression: microglia activation, extracellular microvesicles and microRNA dysregulation. Front. Cell. Neurosci. 9:476. doi: 10.3389/fncel.2015.00476, PMID: 26733805 PMC4681811

[ref10] Caby M.-P. Lankar D. Vincendeau-Scherrer C. Raposo G. Bonnerot C. (2005). Exosomal-like vesicles are present in human blood plasma. Int. Immunol. 17, 879–887. doi: 10.1093/intimm/dxh267, PMID: 15908444

[ref11] Carli V. Wasserman D. Hadlaczky G. Petros N. G. Carletto S. Citi L.. (2020). A protocol for a multicentre, parallel-group, pragmatic randomised controlled trial to evaluate the NEVERMIND system in preventing and treating depression in patients with severe somatic conditions. BMC Psychiatry 20:93. doi: 10.1186/s12888-020-02494-3, PMID: 32122315 PMC7053064

[ref12] Chandrasekar A. Heuvel F. O. Tar L. Hagenston A. M. Palmer A. Linkus B.. (2019). Parvalbumin interneurons shape neuronal vulnerability in blunt TBI. Cereb. Cortex 29, 2701–2715. doi: 10.1093/cercor/bhy139, PMID: 29982364

[ref13] Cohen A. S. Pfister B. J. Schwarzbach E. Grady M. S. Goforth P. B. Satin L. S. (2007). Injury-induced alterations in CNS electrophysiology. Prog. Brain Res. 161, 143–169. doi: 10.1016/s0079-6123(06)61010-8, PMID: 17618975

[ref14] Duarte S. T. Armstrong J. Roche A. Ortez C. Pérez A. O’Callaghan M.. (2013). Abnormal expression of cerebrospinal fluid cation chloride cotransporters in patients with Rett syndrome. PLoS One 8:e68851. doi: 10.1371/journal.pone.0068851, PMID: 23894354 PMC3716803

[ref15] Epsztein J. Represa A. Jorquera I. Ben-Ari Y. Crépel V. (2005). Recurrent mossy fibers establish aberrant Kainate receptor-operated synapses on granule cells from epileptic rats. J. Neurosci. 25, 8229–8239. doi: 10.1523/jneurosci.1469-05.2005, PMID: 16148230 PMC6725550

[ref16] Fuller O. K. Whitham M. Mathivanan S. Febbraio M. A. (2020). The protective effect of exercise in neurodegenerative diseases: the potential role of extracellular vesicles. Cells 9:2182. doi: 10.3390/cells9102182, PMID: 32998245 PMC7599526

[ref17] Gong J. Körner R. Gaitanos L. Klein R. (2016). Exosomes mediate cell contact–independent ephrin-Eph signaling during axon guidance. J. Cell Biol. 214, 35–44. doi: 10.1083/jcb.201601085, PMID: 27354374 PMC4932373

[ref18] Goubert E. Altvater M. Rovira M.-N. Khalilov I. Mazzarino M. Sebastiani A.. (2019). Bumetanide prevents brain trauma-induced depressive-like behavior. Front. Mol. Neurosci. 12:12. doi: 10.3389/fnmol.2019.00012, PMID: 30804751 PMC6370740

[ref19] Hashkavayi A. B. Cha B. S. Lee E. S. Kim S. Park K. S. (2020). Advances in exosome analysis methods with an emphasis on electrochemistry. Anal. Chem. 92, 12733–12740. doi: 10.1021/acs.analchem.0c02745, PMID: 32902258

[ref20] Hsieh T.-H. Lee H. H. C. Hameed M. Q. Pascual-Leone A. Hensch T. K. Rotenberg A. (2016). Trajectory of Parvalbumin cell impairment and loss of cortical inhibition in traumatic brain injury. Cereb. Cortex 27, 5509–5524. doi: 10.1093/cercor/bhw318, PMID: 27909008 PMC6075565

[ref21] Hu D. Yu Z.-L. Zhang Y. Han Y. Zhang W. Lu L.. (2017). Bumetanide treatment during early development rescues maternal separation-induced susceptibility to stress. Sci. Rep. 7:11878. doi: 10.1038/s41598-017-12183-z, PMID: 28928398 PMC5605528

[ref22] Ibrahim S. Hu W. Wang X. Gao X. He C. Chen J. (2016). Traumatic brain injury causes aberrant migration of adult-born neurons in the Hippocampus. Sci. Rep. 6:21793. doi: 10.1038/srep21793, PMID: 26898165 PMC4761898

[ref23] Kahle K. T. Deeb T. Z. Puskarjov M. Silayeva L. Liang B. Kaila K.. (2013). Modulation of neuronal activity by phosphorylation of the K-Cl cotransporter KCC2. Trends Neurosci. 36, 726–737. doi: 10.1016/j.tins.2013.08.006, PMID: 24139641 PMC4381966

[ref24] Kelly K. M. Miller E. R. Lepsveridze E. Kharlamov E. A. Mchedlishvili Z. (2015). Posttraumatic seizures and epilepsy in adult rats after controlled cortical impact. Epilepsy Res. 117, 104–116. doi: 10.1016/j.eplepsyres.2015.09.009, PMID: 26432760

[ref25] Kessler R. C. Aguilar-Gaxiola S. Alonso J. Chatterji S. Lee S. Ormel J.. (2009). The global burden of mental disorders: an update from the WHO world mental health (WMH) surveys*. Epidemiologia e Psichiatr. Soc. 18, 23–33. doi: 10.1017/s1121189x00001421, PMID: 19378696 PMC3039289

[ref26] Ko J. Hemphill M. Yang Z. Sewell E. Na Y. J. Sandsmark D. K.. (2018). Diagnosis of traumatic brain injury using miRNA signatures in nanomagnetically isolated brain-derived extracellular vesicles. Lab a Chip 18, 3617–3630. doi: 10.1039/c8lc00672e, PMID: 30357245 PMC6334845

[ref27] Kourdougli N. Pellegrino C. Renko J. Khirug S. Chazal G. Kukko-Lukjanov T.. (2017). Depolarizing γ-aminobutyric acid contributes to glutamatergic network rewiring in epilepsy. Ann. Neurol. 81, 251–265. doi: 10.1002/ana.24870, PMID: 28074534

[ref28] Kursan S. McMillen T. S. Beesetty P. Dias-Junior E. Almutairi M. M. Sajib A. A.. (2017). The neuronal K+Cl− co-transporter 2 (Slc12a5) modulates insulin secretion. Sci. Rep. 7:1732. doi: 10.1038/s41598-017-01814-0, PMID: 28496181 PMC5431760

[ref29] Lange R. T. Lippa S. M. French L. M. Bailie J. M. Gartner R. L. Driscoll A. E.. (2020). Long-term neurobehavioural symptom reporting following mild, moderate, severe, and penetrating traumatic brain injury in U.S. military service members. Neuropsychol. Rehabilitation 30, 1762–1785. doi: 10.1080/09602011.2019.1604385, PMID: 31003592

[ref30] Li J.-J. Ji R. Shi Y.-Q. Wang Y.-Y. Yang Y.-L. Dou K.-F. (2012). Changes in expression of the chloride homeostasis-regulating genes, KCC2 and NKCC1, in the blood of cirrhotic patients with hepatic encephalopathy. Exp. Ther. Med. 4, 1075–1080. doi: 10.3892/etm.2012.721, PMID: 23226777 PMC3494113

[ref31] Liu Y. Cao X. (2016). Organotropic metastasis: role of tumor exosomes. Cell Res. 26, 149–150. doi: 10.1038/cr.2015.153, PMID: 26704450 PMC4746605

[ref32] Löscher W. Kaila K. (2022). CNS pharmacology of NKCC1 inhibitors. Neuropharmacology 205:108910. doi: 10.1016/j.neuropharm.2021.108910, PMID: 34883135

[ref33] Louis D. N. Ohgaki H. Wiestler O. D. Cavenee W. K. Burger P. C. Jouvet A.. (2007). The 2007 WHO classification of tumours of the central nervous system. Acta Neuropathol. 114, 97–109. doi: 10.1007/s00401-007-0243-4, PMID: 17618441 PMC1929165

[ref34] Ludwig A. Li H. Saarma M. Kaila K. Rivera C. (2003). Developmental up-regulation of KCC2 in the absence of GABAergic and glutamatergic transmission. Eur. J. Neurosci. 18, 3199–3206. doi: 10.1111/j.1460-9568.2003.03069.x, PMID: 14686894

[ref35] Luscher B. Fuchs T. (2015). Chapter five GABAergic control of depression-related brain states. Adv. Pharmacol. 73, 97–144. doi: 10.1016/bs.apha.2014.11.003, PMID: 25637439 PMC4784429

[ref36] Medina I. Friedel P. Rivera C. Kahle K. T. Kourdougli N. Uvarov P.. (2014). Current view on the functional regulation of the neuronal K+-Cl− cotransporter KCC2. Front. Cell. Neurosci. 8:27. doi: 10.3389/fncel.2014.00027, PMID: 24567703 PMC3915100

[ref37] Meyer K. Helmick K. Doncevic S. Park R. (2008). Severe and penetrating traumatic brain injury in the context of war. J. Trauma Nurs. 15, 185–189. doi: 10.1097/01.jtn.0000343324.55087.de, PMID: 19092508

[ref38] Patel T. P. Ventre S. C. Geddes-Klein D. Singh P. K. Meaney D. F. (2014). Single-neuron NMDA receptor phenotype influences neuronal rewiring and reintegration following traumatic injury. J. Neurosci. 34, 4200–4213. doi: 10.1523/jneurosci.4172-13.2014, PMID: 24647941 PMC3960464

[ref39] Peeters W. van den Brande R. Polinder S. Brazinova A. Steyerberg E. W. Lingsma H. F.. (2015). Epidemiology of traumatic brain injury in Europe. Acta Neurochir. 157, 1683–1696. doi: 10.1007/s00701-015-2512-7, PMID: 26269030 PMC4569652

[ref40] Pellegrino C. Gubkina O. Schaefer M. Becq H. Ludwig A. Mukhtarov M.. (2011). Knocking down of the KCC2 in rat hippocampal neurons increases intracellular chloride concentration and compromises neuronal survival. J. Physiol. 589, 2475–2496. doi: 10.1113/jphysiol.2010.203703, PMID: 21486764 PMC3115820

[ref41] Perry D. C. Sturm V. E. Peterson M. J. Pieper C. F. Bullock T. Boeve B. F.. (2016). Association of traumatic brain injury with subsequent neurological and psychiatric disease: a meta-analysis. J. Neurosurg. 124, 511–526. doi: 10.3171/2015.2.jns14503, PMID: 26315003 PMC4751029

[ref42] Pin-Barre C. Constans A. Brisswalter J. Pellegrino C. Laurin J. (2017). Effects of high- versus moderate-intensity training on neuroplasticity and functional recovery after focal ischemia. Stroke 48, 2855–2864. doi: 10.1161/strokeaha.117.017962, PMID: 28904232

[ref43] Pulliam L. Sun B. Mustapic M. Chawla S. Kapogiannis D. (2019). Plasma neuronal exosomes serve as biomarkers of cognitive impairment in HIV infection and Alzheimer’s disease. J. NeuroVirology 25, 702–709. doi: 10.1007/s13365-018-0695-4, PMID: 30610738 PMC7372698

[ref44] Quek C. Hill A. F. (2017). The role of extracellular vesicles in neurodegenerative diseases. Biochem. Biophys. Res. Commun. 483, 1178–1186. doi: 10.1016/j.bbrc.2016.09.090, PMID: 27659705

[ref45] Rivera C. Voipio J. Payne J. A. Ruusuvuori E. Lahtinen H. Lamsa K.. (1999). The K+/cl− co-transporter KCC2 renders GABA hyperpolarizing during neuronal maturation. Nature 397, 251–255. doi: 10.1038/16697, PMID: 9930699

[ref46] Roddy D. W. Farrell C. Doolin K. Roman E. Tozzi L. Frodl T.. (2019). The Hippocampus in depression: more than the sum of its parts? Advanced hippocampal substructure segmentation in depression. Biol. Psychiatry 85, 487–497. doi: 10.1016/j.biopsych.2018.08.021, PMID: 30528746

[ref47] Sankar R. Mazarati A. (2010). Neurobiology of depression as a comorbidity of epilepsy. Epilepsia 51:81. doi: 10.1111/j.1528-1167.2010.02867.x, PMID: 21415938 PMC3056239

[ref48] Santhakumar V. Ratzliff A. D. H. Jeng J. Toth Z. Soltesz I. (2001). Long-term hyperexcitability in the hippocampus after experimental head trauma. Ann. Neurol. 50, 708–717. doi: 10.1002/ana.1230, PMID: 11761468

[ref49] Scala C. D. Tessier M. Sapet C. Poulhes F. Sicard F. Zelphati O.. (2019). A new polymer-based approach for in vivo transfection in postnatal brain. J. Neurosci. Methods 311, 295–306. doi: 10.1016/j.jneumeth.2018.11.004, PMID: 30408559

[ref50] Sharma P. Mesci P. Carromeu C. McClatchy D. R. Schiapparelli L. Yates J. R.. (2019). Exosomes regulate neurogenesis and circuit assembly. Proc. Natl. Acad. Sci. 116, 16086–16094. doi: 10.1073/pnas.1902513116, PMID: 31320591 PMC6689941

[ref51] Shulga A. Magalhães A. C. Autio H. Plantman S. di Lieto A. Nykjær A.. (2012). The loop diuretic bumetanide blocks posttraumatic p75NTR upregulation and rescues injured neurons. J. Neurosci. 32, 1757–1770. doi: 10.1523/jneurosci.3282-11.2012, PMID: 22302815 PMC6703341

[ref52] Shulga A. Thomas-Crusells J. Sigl T. Blaesse A. Mestres P. Meyer M.. (2008). Posttraumatic GABAA-mediated [Ca2+]i increase is essential for the induction of brain-derived neurotrophic factor-dependent survival of mature central neurons. J. Neurosci. 28, 6996–7005. doi: 10.1523/jneurosci.5268-07.2008, PMID: 18596173 PMC6670975

[ref53] Sloviter R. S. (2008). Hippocampal epileptogenesis in animal models of mesial temporal lobe epilepsy with hippocampal sclerosis: the importance of the “latent period” and other concepts. Epilepsia 49, 85–92. doi: 10.1111/j.1528-1167.2008.01931.x, PMID: 19087122

[ref54] Tiwari S. Kumar V. Randhawa S. Verma S. K. (2021). Preparation and characterization of extracellular vesicles. Am. J. Reprod. Immunol. 85:e13367. doi: 10.1111/aji.13367, PMID: 33118232

[ref55] Tunc-Ozcan E. Peng C.-Y. Zhu Y. Dunlop S. R. Contractor A. Kessler J. A. (2019). Activating newborn neurons suppresses depression and anxiety-like behaviors. Nat. Commun. 10:3768. doi: 10.1038/s41467-019-11641-8, PMID: 31434877 PMC6704083

[ref56] Vigil F. A. Bozdemir E. Bugay V. Chun S. H. Hobbs M. Sanchez I.. (2019). Prevention of brain damage after traumatic brain injury by pharmacological enhancement of KCNQ (Kv7, “M-type”) K+ currents in neurons. J. Cereb. Blood Flow Metab. 40, 1256–1273. doi: 10.1177/0271678x19857818, PMID: 31272312 PMC7238379

